# Prognosis in Albuminuric Retinitis

**Published:** 1901-12-28

**Authors:** 


### PROGNOSIS IN ALBUMINURIC RETINITIS.

There are two points to be considered in discussing the outlook in any case of albuminuric retinitis, first the prognosis as to vision ; and, secondly, the prognosis as to life. Mr. Walter H. H. Jessop [1] says in regard to the first point, that patients rarely, if ever, become blind from the changes in the retina. Even the serious diminution of vision which occurs in the retinitis associated with pregnancy may be completely recovered from, and in all the cases he has seen, and also in the true retinitis associated with acute nephritis and scarlet fever, the impaired vision has tended to become very much better. When the vision is affected in cases of granular kidney, the impairment is generally gradual and good vision may persist to the end of life even in cases of extreme retinal disturbance. Indeed, if vision be lost in such cases it is as a rule due to the advent of uræmia.

As to the bearing of the existence of uræmic retinitis upon prognosis in cases of granular kidney, this is very serious owing to the fact that such retinitis occurs at a late stage of the disease. Speaking generally it would appear that very few cases live beyond two years after the retinitis has been diagnosed. We must bear in mind, however, that as the acuity of vision is seldom much affected at first it may have happened that the disease may have been well advanced before it was discovered in many of the cases on which this statement is founded, and hence that the prognosis of an accidentally discovered retinitis might not of necessity be so serious as is here suggested. Coming to Mr. Jessop's personal experience on the subject we find that he has never known a patient live more than four years, and only two as long as that, and that most patients die within a year after the diagnosis is made. Finally, we must bear in mind that there exists a uræmic amaurosis which is quite a different thing from albuminuric retinitis, although both may occur in the course of the same disease, and, indeed, in the same patient, so that defective vision in cases of albuminuria must not be taken without examination to mean retinitis.

[1] Practitioner, Dec., 1901.

